# Rapid and complete inactivation of SARS-CoV-2 by ultraviolet-C irradiation

**DOI:** 10.1038/s41598-020-79600-8

**Published:** 2020-12-30

**Authors:** Nadia Storm, Lindsay G. A. McKay, Sierra N. Downs, Rebecca I. Johnson, Dagnachew Birru, Marc de Samber, Walter Willaert, Giovanni Cennini, Anthony Griffiths

**Affiliations:** 1 National Emerging Infectious Diseases Laboratories, Boston University School of Medicine, 620 Albany Street, Boston, MA 02118 USA; 2 Signify Research, 1 Charles Park, 2nd Fl, Cambridge, MA 02142 USA; 3 Signify Research, High Tech Campus 7, 5656 AE Eindhoven, the Netherlands

**Keywords:** Microbiology, Virology, SARS-CoV-2

## Abstract

The severe acute respiratory syndrome coronavirus 2 (SARS-CoV-2) pandemic has devastated global public health systems and economies, with over 52 million people infected, millions of jobs and businesses lost, and more than 1 million deaths recorded to date. Contact with surfaces contaminated with droplets generated by infected persons through exhaling, talking, coughing and sneezing is a major driver of SARS-CoV-2 transmission, with the virus being able to survive on surfaces for extended periods of time. To interrupt these chains of transmission, there is an urgent need for devices that can be deployed to inactivate the virus on both recently and existing contaminated surfaces. Here, we describe the inactivation of SARS-CoV-2 in both wet and dry format using radiation generated by a commercially available Signify ultraviolet (UV)-C light source at 254 nm. We show that for contaminated surfaces, only seconds of exposure is required for complete inactivation, allowing for easy implementation in decontamination workflows.

## Introduction

Towards the end of 2019, an outbreak of life-threatening pneumonia caused by a novel betacoronavirus occurred in the Hubei Province of China^1^. The virus, named severe acute respiratory syndrome coronavirus 2 (SARS-CoV-2), has since spread across the world at an alarming rate to cause a debilitating and ongoing pandemic, with only a few islands not reporting any cases to date. While SARS-CoV-2 is thought to be of zoonotic origin^2^, intense and extensive human-to-human transmission has mainly been driven by the inhalation of respiratory droplets and virus-bearing particles spread through the air^3^, or by contact with surfaces contaminated with settled droplets^4^. Although academic institutions and pharmaceutical organizations worldwide have banded together to develop countermeasures against the virus, there are still no licensed vaccines or therapeutics available. The disruption of transmission chains is therefore crucial for managing the outbreak and preventing additional infections.

Ultraviolet (UV) irradiation is an extensively tested, widely used and effective no-contact method for inactivating viral pathogens^5–7^. There are three types of UV, including UV-A (315–400 nm), UV-B (280–315 nm) and UV-C (100–280 nm), of which UV-C is most commonly employed in germicidal applications. At a wavelength of 254 nm, viral inactivation can be attributed to direct UV-C light absorption and photochemical damage to nucleic acid, leading to the disruption of viral replication^8^. Despite its wide use, limited data exists on the effectiveness of UV-C on inactivating wet and dried SARS-CoV-2 on contaminated surfaces. In particular, the efficacy of UV-C for inactivating SARS-CoV-2 in fluids needs to be determined, as the UV absorbance characteristics of fluid constituents may influence the dose required to achieve complete viral inactivation.

In this paper, we describe the complete and rapid inactivation of SARS-CoV-2 in both wet and dried droplets using 254 nm UV-C irradiation. Our results suggest that UV-C is an affordable and effective tool for preventing SARS-CoV-2 contact transmission that can easily be deployed to manage the coronavirus disease outbreak.

## Results and discussion

### Estimation of viral decay time

**Figure 1:**
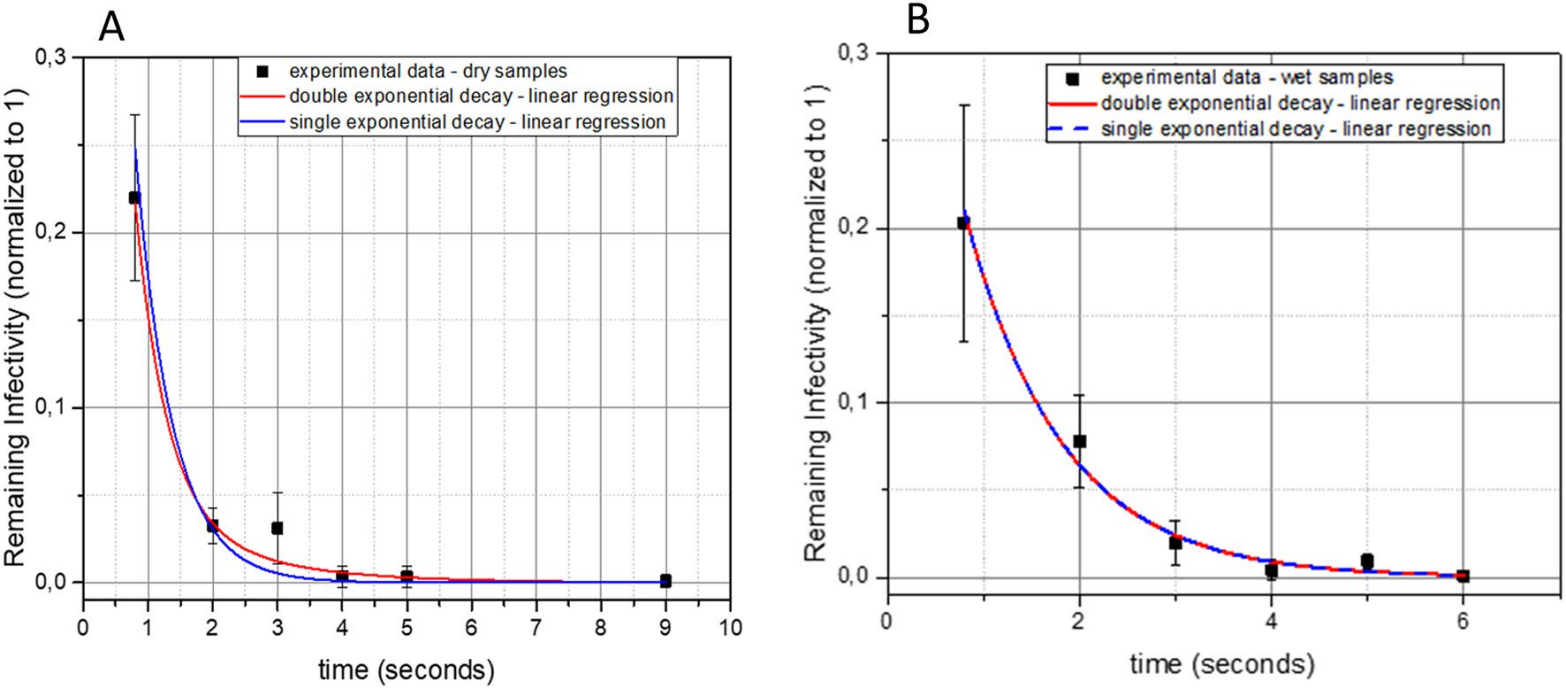
Reduction in infectivity of SARS-CoV-2 after exposure to UV-C irradiation. The virus was exposed to UV-C as dried droplets (**A**) or wet droplets (**B**). Each set of data (dry samples and wet samples) shows a decrease of the remaining infectivity as a function of time, normalized to 1. Blue lines indicate single exponential decay functions while red lines indicate double exponential decay functions.

To examine the inactivation efficacy of UV-C on SARS-CoV-2, virus was applied to plastic tissue culture dishes and exposed to UV radiation as either wet or dried droplets for varying amounts of time ranging from 0.8 to 120 s. Under a UV-C irradiance of 0.849 mW/cm^2^, partial inactivation occurred from 0.8 s of exposure, while SARS-CoV-2 virus infectivity was reduced to below detectable levels in as few as 9 s for dried virus (Table 1; Fig. 1A) and 4 s for wet virus (Table 1; Fig. 1B).

**Table 1:** Reduction in viral titer (PFU/ml) at different irradiation times.

Seconds	0.8	2	3	4	5	6	9	120
Wet virus	517	170	53	13	13	2	ND	NT
Wet virus control	2150	2100	2167	2267	1450	1700	1550	NT
Percent reduction	75.9	91.9	97.6	99.4	99.1	99.9	> 99.9	N/A
Dry virus	85	39	8	1	ND	ND	ND	ND
Dry virus control	513	523	503	563	613	550	563	420
Percent reduction	83.4	92.5	98.4	99.8	> 99.9	> 99.9	> 99.9	> 99.9

*PFU/ml* plaque-forming units per milliliter, *ND* not detected, *NT* not tested, *N/A* not applicable.

Virus inactivation by UV light is expected to be an exponential process^9^. Therefore, to estimate the decay time, we used linear regression methods with single and double exponential decay functions (Fig. 1). The single exponential decay function has the form ${\mathrm{y}}= {\mathrm{e}}^{-t/\uptau }$, while the double exponential function has the form $\mathrm{y}= {\left(1-f\right)\mathrm{e}}^{-t/{\uptau }_{1}}+f{\mathrm{e}}^{-t/{\uptau }_{2}}$. τ, τ_1_ and τ_2_ are the decay times of the linear regressions. In case of double exponential decay, *f* is the fraction of the viruses that survive the first decay. For the analysis, data points were normalized so that the initial condition *t* = 0 corresponds to 100% infectivity with no irradiance.

In the linear regression of dried droplets, the reduced *χ*^2^ for double exponential decay (0.36) was lower than the one corresponding to single exponential decay (0.52). The R^2^ for double exponential was higher than the R^2^ of the single exponential. Hence, we used the double exponential decay to estimate the decay times, obtaining ${\uptau }_{1}=0.48\pm 0.09\mathrm{ s}$, and ${\uptau }_{2}= 1.60\pm 1.17\mathrm{ s}$.

In case of wet droplets, we observed the opposite: the χ^2^ for the double exponential (1.0) was higher than the one corresponding to the single exponential (0.8). The R^2^ for the double exponential and the single exponential was the same (0.9). We therefore used the single exponential decay as a best fit of the data to estimate the decay time, translating into an average decay time of $\uptau =1.0\pm 0.1\mathrm{ s}$. Within one standard deviation, the decay times of wet and dried droplets are congruent. This is most likely due to the limited resolution of the measurements. In addition, this indicates that given the observation limits, UV-C absorption by media constituents did not significantly affect virus inactivation at a wavelength of 254 nm.

It should be noted that the experiments for this study were performed under specific and controlled conditions. Factors such as humidity, textured surfaces and the presence of dust and other particles may reduce the effectiveness of UV-C and influence the dose required to achieve complete viral inactivation^6^. It is also important to consider the composition of respiratory droplets when evaluating the effectiveness of 254 nm UV-C irradiation. Droplets are likely to be in solvent with a variety of other biological fluids such as respiratory mucus (phlegm) which may include viral glycoproteins, and UV-C absorption of these fluids and particles may result in a reduction in viral inactivation efficiencies. The results obtained in this study should therefore be interpreted as the minimum dose of radiation required to achieve viral inactivation.

A recent study looked at the combined effectiveness of UV-A/UV-C irradiation of SARS-CoV-2 in comparison to UV-A or UV-C alone^10^. The study found that UV-A was a poor means of inactivating SARS-CoV-2 in comparison to inactivation by 1.94 mW/cm^2^ UV-C. Of note, our results were generated using a lower dose of UV-C (0.849 mW/cm^2^) and a quicker time (4 – 9 s rather than 9 min with UV-C alone). This supports our observations showing the minimum required UV-C irradiance and time needed for complete SARS-CoV-2 inactivation. Another recent study investigated the effectiveness of light-emitting diode (LED) UV-C irradiation (280 nm)^11^ in inactivating SARS-CoV-2 in wet droplets. Similar to our findings, these results indicate inactivation of wet virus within 10 s at a UV dose of 3.75 mW/cm^2^. Using linear regression methods of analysis, our data provides a comprehensive overview of viral decay as a function of time for both wet (4 s) and dried (9 s) virus. Given the extended survival of SARS-CoV-2 on surfaces such as bank notes, glass and stainless steel^14^, the ability of countermeasures such as UV devices to inactivate virus on previously contaminated surfaces is crucial and was demonstrated using dried virus in our study.

Although it was beyond the scope of this study, future studies should address the effects of humidity, surface conformation and the natural matrices in which the virus may exist on the required UV-C inactivation doses. Direct exposure of the skin and eyes to 254 nm UV-C light can present a serious health hazard, such as corneal irritation and burns^12,13^ and as such, 254 nm UV-C light should only be used with proper training or where people are not at risk of being exposed. Forthcoming studies will explore the viral inactivation effectiveness of far UV-C wavelengths (207–222 nm) that have been proposed to be a safer alternative to 254 nm UV-C light^7^.

Although several techniques exist for inactivating SARS-CoV-2, the lack of proven effective tools and interventions have allowed for the unmanageable spread of the virus in the human population. Our results show that UV-C is a powerful tool that can be applied extensively in a wide range of public institutions including hospitals, nursing homes, workplaces, schools, airports and shopping centers to disinfect contaminated equipment and surfaces to prevent and reduce SARS-CoV-2 contact transmission.

## Methods

### UV-C device

**Figure 2:**
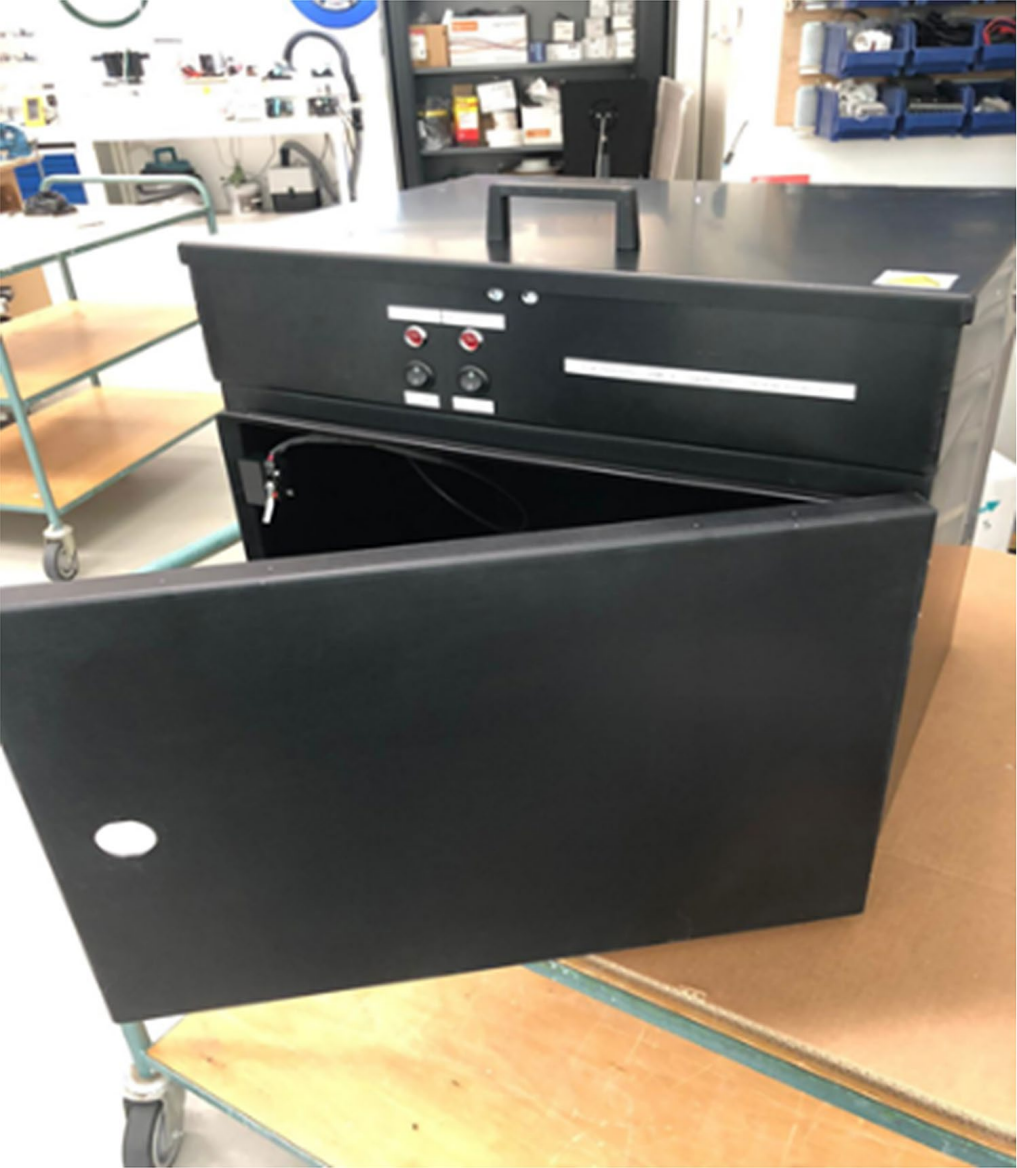
Prototype UV-C test apparatus.

A test apparatus was designed, optimized, fabricated and calibrated to enable accurate and controlled UV-C treatment of test samples (Fig. 2). A collimated beam setup was fabricated based on a dual chamber construction. The top chamber contains the UV-C light source, the electronic driver, and a shutter system to control the exposure times of the samples while keeping the lamp output stable.

**Figure 3:**
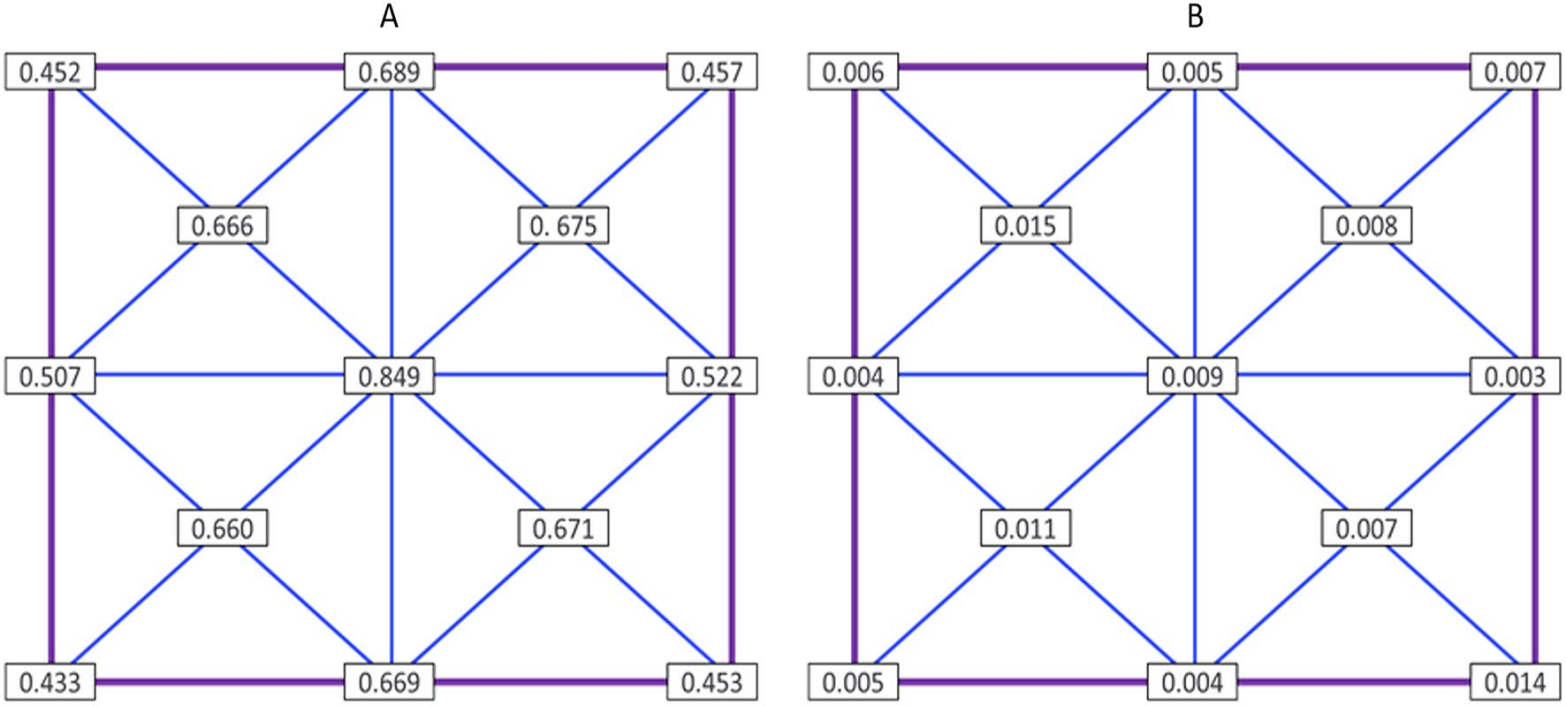
Average values of irradiance (**a**) and standard deviations (**b**) obtained from testing three lamps at given points within the UV-C device (mW/cm^2^).

Samples were treated in the bottom chamber using deep UV-C light generated with a classical Mercury type TUV PLL 35 W light source, generating a peak wavelength at 254 nm. Multiple sensor-based safety measures were applied to protect the user against incidental exposure to the UV-C radiation. The irradiance level for three different lamps inside the treatment chamber was measured using a calibrated UV-C sensor system (Spectroradiometer GL Optic Spectis 5.0 Touch with detector GL Opti Probe 5.1.50), which provided irradiance patterns and levels shown in Fig. 3 from which optimal treatment locations could be deduced.

### Virus inactivation procedures

All experiments were performed in the biosafety level 4 laboratory of the National Emerging Infectious Diseases Laboratories of Boston University. A volume of 100 µl SARS-CoV-2 (7.33 × 10^3^ PFU/ml) (USA/WA1-2020)^15^ was plated onto the surface of 60 mm plastic tissue culture dishes (TPP) in 5 µl aliquots. The virus was allowed to dry for approximately 2 h on a subset of the dishes while the rest were processed immediately in the prototype UV-C device. Briefly, a pair of dishes (one to be treated and one control wrapped tightly in aluminum foil) were placed in the center of the device at an irradiance level of 0.849 mW/cm^2^, and towards the side of the UV-C device, respectively. The dishes were UV-C-treated for either 0.8, 2, 3, 4, 5, 6, 9, 15, 30 or 120 s, with each treatment time tested in triplicate. Dishes containing dried virus were treated in the same manner. Following treatment, the wet and dried virus were resuspended in 1.9 ml or 2 ml, respectively, of high glucose Dulbecco's Modified Eagle Medium (DMEM)(Gibco) containing 0.04 mM phenol red, 1 × antibiotic–antimycotic (Gibco), 1 × non-essential amino acids (Gibco), 1 × GlutaMAX-I (Gibco), 1 mM sodium pyruvate (Gibco) and 2% fetal bovine serum (FBS)(Gibco). The resuspended virus was then serially diluted from 1 × 10^0^ to 1 × 10^–2.5^ using half-logarithmic dilutions. A back-titration of the virus was included for each experiment.

### Confirmation of virus inactivation by plaque assay

Vero E6 cells maintained in high glucose DMEM (Gibco) supplemented with 1 × GlutaMAX-I, 1 mM sodium pyruvate, 10% FBS (Gibco) and 1 × non-essential amino acids (Gibco) were seeded into 6-well CellBIND plates (Corning) at a density of 8.0 × 10^5^ cells per well. The cells were incubated at 37 °C and 5% CO_2_ overnight. The media was removed from each well and 200 µl of each dilution prepared from resuspended virus was added to the respective wells of a 6-well plate. One well containing only DMEM with 2% FBS was included as a control on each plate. A back-titer of the virus used to prepare the 60 mm dishes was performed in triplicate by inoculating each well of a 6-well plate with 1 × 10^–2^ to 1 × 10^–6^ dilutions of the virus, respectively. Plates were incubated at 37 °C and 5% CO_2_ for 1 h with intermittent rocking. Cells were then overlaid with 2 ml of a 1:1 solution of 2.5% Avicel RC-591 (DuPont Nutrition and Health) and 2 × Temin's Modified Eagle Medium (Gibco) without phenol red, supplemented with 10% FBS (Gibco), 2 × antibiotic–antimycotic (Gibco) and 2 × GlutaMAX-I (Gibco). The cells were incubated at 37 °C and 5% CO_2_ for 2 days. Plates were fixed in 10% neutral buffered formalin (ThermoFisher Scientific), followed by staining with 0.2% Gentian Violet (Ricca Chemical) in 10% neutral buffered formalin. The number of plaques per virus dilution were determined by eye and used to calculate the titer of the virus using the following formula:

$$ {\text{Virus titer in PFU}}/{\text{ml}} = {\text{Number of plaques }}/\left( {{\text{virus dilution in well}} \times {\text{volume plated in ml}}} \right) $$

### Statistical analysis

The statistical package Microcal Origin was used to analyze the data. A detailed explanation of the statistical methods used is provided with the results.

## Data Availability

Additional data supporting the findings of this study are available from the corresponding author upon request.
